# Emergence and loss of spliceosomal twin introns

**DOI:** 10.1186/s40694-017-0037-y

**Published:** 2017-10-06

**Authors:** Michel Flipphi, Norbert Ág, Levente Karaffa, Napsugár Kavalecz, Gustavo Cerqueira, Claudio Scazzocchio, Erzsébet Fekete

**Affiliations:** 1 Department of Biochemical Engineering, University of Debrecen, Debrecen, 4032 Hungary; 2 Broad Institute of MIT and Harvard, Cambridge, MA USA; 3 Department of Microbiology, Imperial College London, London, UK; 4 Institut de Biologie Intégrative de la Cellule, CEA/CNRS, Université Paris-Saclay UMR, 9198 Orsay, France

**Keywords:** Spliceosomal twin introns, Spliceosomal intron evolution, Intron gain, Intron loss, Molecular phylogenetics, Pezizomycotina, *Aspergillus nidulans*

## Abstract

**Background:**

In the primary transcript of nuclear genes, coding sequences—exons—usually alternate with non-coding sequences—introns. In the evolution of spliceosomal intron–exon structure, extant intron positions can be abandoned and new intron positions can be occupied. Spliceosomal twin introns (“stwintrons”) are unconventional intervening sequences where a standard “internal” intron interrupts a canonical splicing motif of a second, “external” intron. The availability of genome sequences of more than a thousand species of fungi provides a unique opportunity to study spliceosomal intron evolution throughout a whole kingdom by means of molecular phylogenetics.

**Results:**

A new stwintron was encountered in *Aspergillus nidulans* and *Aspergillus niger*. It is present across three classes of Leotiomyceta in the transcript of a well-conserved gene encoding a putative lipase (*lipS*). It occupies the same position as a standard intron in the orthologue gene in species of the early divergent classes of the Pezizomycetes and the Orbiliomycetes, suggesting that an internal intron has appeared within a pre-extant intron. On the other hand, the stwintron has been lost from certain taxa in Leotiomycetes and Eurotiomycetes at several occasions, most likely by a mechanism involving reverse transcription and homologous recombination. Another ancient stwintron present across whole Pezizomycotina orders—in the transcript of the bifunctional biotin biosynthesis gene *bioDA*—occurs at the same position as a standard intron in many species of non-Dikarya. Nevertheless, also the *bioDA* stwintron has disappeared from certain lineages within the taxa where it occurs, i.e., Sordariomycetes and Botryosphaeriales. Intriguingly, only the internal intron was lost from the Sordariomycetes *bioDA* stwintron at all but one occasion, leaving a standard intron in the same position, while where the putative lipase stwintron was lost, no intronic sequences remain.

**Conclusions:**

Molecular phylogeny of the peptide product was used to monitor the existence and fate of a stwintron in the transcripts of two neatly defined fungal genes, encoding well conserved proteins. Both defining events—stwintron emergence and loss—can be explained with extant models for intron insertion and loss. We thus demonstrate that stwintrons can serve as model systems to study spliceosomal intron evolution.

**Electronic supplementary material:**

The online version of this article (doi:10.1186/s40694-017-0037-y) contains supplementary material, which is available to authorized users.

## Background

In the primary transcript of nuclear genes, coding sequences—exons—usually alternate with non-coding sequences—introns. The latter are removed and former are joined by means of splicing to create the mRNA ORF that translates into the functional peptide product (for a review, [1]). In the evolution of intron–exon structures of transcripts of nuclear genes, extant intron positions can be abandoned and new intron positions can be occupied. Loss and gain of introns are processes that can take place at different rates leading to three distinct modes of intron dynamics [2]. In most present day species, intron loss and -gain balance each other overall. The availability of complete genome sequences of more than a thousand species of fungi provides a unique opportunity to study intron evolution in conserved genes throughout a whole kingdom.

Spliceosomal twin introns (“stwintrons”) are complex intervening sequences that consist of a canonical U2 intron within another canonical U2 intron, arranged in such a way that one of these (the “internal” intron) interrupts one of the conserved domain sequences of the second one (the “external” intron)—i.e., the donor at the 5′ splice site, the acceptor at the 3′ splice site or the sequence around the lariat branch point adenosine. A consequence of this intron nestling is that the external intron is only functional *after* excision of the internal intron and, hence, consecutive splicing reactions are necessary to generate a mature mRNA. We have characterised stwintrons in different fungal species where the internal intron interrupts the donor of the external intron between the first and the second, or between the second and third nucleotides—[D1,2] and [D2,3] stwintrons, respectively [3–5]. The two-step splicing process resulting in stwintron excision demonstrated that splice sites pair via intron definition (cf. [6]) in filamentous ascomycota (Pezizomycotina). For the [D1,2] stwintron we found in the alternative oxidase transcript of *Helminthosporium solani*, we demonstrated a complex structure that could undergo two alternative pathways of sequential splicing. An internal intron could be envisaged as interrupting the donor sequence *or* the acceptor sequence of an external intron. In every case where a [D1,2] stwintron is extant (which implies that the complex intervening sequence starts with two successive Gs at its 5′ terminus), a stwintron in which the acceptor sequence of the external intron is disrupted between the penultimate and ultimate nucleotides—an [A2,3] stwintron—will occur every time that the 5′ base of the downstream exon is also a G. This complex arrangement results in two mutually exclusive, alternative ways to obtain the same mature mRNA via distinct splicing intermediates. Such an alternatively spliced [D1,2]/[A2,3] stwintron was also found recently in the gene encoding a putative multidrug efflux pump in some taxa of Pezizomycotina [5].

Molecular phylogeny of orthologue proteins encoded by genes that harbour stwintrons and the evolutionary fate of that stwintron may contribute to our understanding of the mechanisms by which spliceosomal introns come into existence and by which they cease to exist. In previous publications, we identified and characterised fungal stwintrons that we had serendipitously encountered while pursuing other work. Unfortunately, all but one of these occur exclusively in one or in a few closely related species. Only the [D2,3] stwintron in the biotin-biosynthetic bifunctional gene *bioDA* was found across a complete class of fungi, the Sordariomycetes. This stwintron thus appears evolutionary stable: loss of any of the internal splice sites will inevitably result in the inability to properly remove the complete intervening sequence from the primary transcript and hence, in biotin auxotrophy. Nevertheless in the *bioDA* gene of *Nectria haematococca,* a standard U2 intron is present at the position occupied by a [D2,3] stwintron in other Sordariomycetes.

To facilitate molecular and mutational study of mechanisms involved in the emergence and disappearance of spliceosomal introns using stwintrons as model systems, we initiated a search for *bona fide* stwintrons in the amenable species *Aspergillus nidulans*, in which molecular- and classical genetic tools are readily developed [7–13]. We devised a crude informatics tool that enabled us to identify putative stwintrons in whole genome sequences, the principle and basics of which were described in detail in [5]. Here, we describe a stwintron we have uncovered in a putative lipase gene after a preliminary screen of the *A. nidulans* genome sequences for [D1,2] stwintrons. We also experimentally verified its presence in *Aspergillus niger*. This stwintron is present across three classes of Pezizomycotina in the transcript of a well-conserved gene without paralogues of note, sufficiently ancient to monitor its emergence and evolution.

## Results

### A new [D1,2] stwintron in *Aspergillus nidulans* and *Aspergillus niger*

**Fig. 1:**
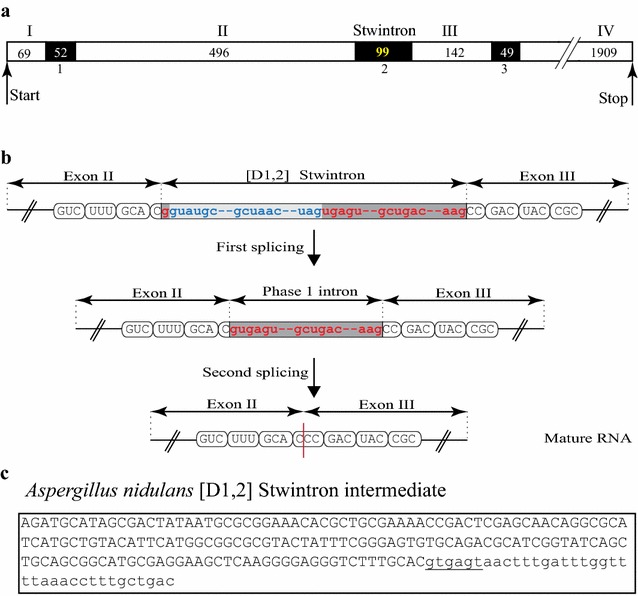
The [D1,2] stwintron of the putative lipase encoding gene of *A. nidulans*. **a** Depicts the determined *A. nidulans lipS* intron–exon structure with the exact sizes of the alternating exons and intervening sequences given. The stwintron is the second intervening sequence. **b** Schematically the structure of the phase-1 stwintron that splits the CCC codon of Pro189, and the two consecutive splicing events necessary to remove it. Exonic sequences are printed in capitals, grouped as the consecutive codons (Val186–Phe187–Ala188–Pro189–Asp190–Tyr191–Arg192). The internal intron is marked by the lighter grey bar; its 5′-donor-, lariat branch point domain- and 3′-acceptor sequences are printed in blue lettering. The external intron is marked by the darker grey bar; its 5′-donor-, lariat branch point domain- and 3′-acceptor sequences are printed in red lettering. **c** The existence of the splicing intermediate from which the internal intron has been removed by the first excision, has been confirmed experimentally (GenBank MF612152). The newly formed donor splice site sequence of the retained external intron, (5′-gugagu), is underlined

An informatics method to detect putative [D1,2] stwintrons has been detailed elsewhere as auxiliary methodology [5]. We screened the genome sequences of *A. nidulans* (whole genome shotgun master accession AACD00000000: annotated scaffolds CH236920–CH236936) and detected, amongst dozens of others, a candidate [D1,2] stwintron at locus AN7524. This locus is predicted by auto-annotation to harbour two intervening sequences. At 69 nt from the ATG, there would be a small canonical intron of 52 nt (5′-GUACGU—33-nt—ACUGAC—4-nt—CAG). 497 nt further downstream, a 289-nt-long, phase 2 intron (5′-GUAUGC—268-nt—GCUAAU—6-nt—CAG) would split a CGA (Arg) codon, while the mature messenger would code for a peptide of 824 amino acids. However, our stwintron search tool suggested the presence of a [D1,2] stwintron at the 5′ end of this large auto-annotated second intron, of which the proposed donor sequence would actually serve as the donor of the 53-nt long internal intron (5′-GUAUGC—33-nt—GCUAAC—5-nt—UAG). This internal intron would disrupt the donor of a 46-nt long external intron between the first and second nt (5′-G|UGAGU—25-nt—GCUGAC—6-nt—AAG). As shown in the splicing scheme depicted in Fig. 1, the 99-nt long [D1,2] stwintron would be excised by consecutive U2 splicing reactions and would split a CCC (Pro) codon behind the first nt. At the end of the large theoretical auto-annotated intron (289-nt), we predicted the presence of a small U2 intron of 49 nt (5′-GUGAGU—28-nt—GCUAAU—6-nt—CAG) using the closest canonical donor sequence available upstream of the lariat branchpoint domain/acceptor couple. Strict application of intron definition (cf. [6]) for the stwintron at the second intron position and the downstream third intron leaves an exon of 142 nt in between, unrecognised by auto-annotation. According to our predicted intron–exon structure of locus AN7524 (Fig. 1a), there would thus be four exons.

The *A. nidulans* gene is best expressed growing on complete medium as compared with minimal medium conditions used for high-throughput RNA sequencing (JBrowse module at AspGD; [8]). Nevertheless, there are no RNASeq reads from which the complete stwintron was absent nor reads from which the large auto-annotated intron sequence was removed (data not shown). We therefore sought to confirm excision of all three intervening sequences we predicted by sequencing of cDNA generated from total RNA isolated from a 16-h cultivation in complete medium (see “Methods” section for details). The cDNA (GenBank Accession number MF612150) specifies a reading frame of 2616 nt that codes for a peptide of 871 amino acids. The first intron separates the codons for His23 and Tyr24, the [D1,2] stwintron splits the codon for Pro189 while the third intron splits the codon for Ser236 (Fig. 1a). Next, we confirmed the existence of the predicted splicing intermediate of the [D1,2] stwintron (Fig. 1b) with our previously published RT-PCR strategy using a reverse primer that terminally overlaps the 3′ distal junction with the predicted external intron to avoid amplification off fully spliced RNA. Upon cloning and sequencing, the smaller amplified fragment was shown to lack the predicted internal intron (53 nt) with the 5′-GUGAGU donor of the external intron reconstructed (Fig. 1c). We deposited the determined sequence of this splicing intermediate at GenBank (MF612152).

**Fig. 2:**
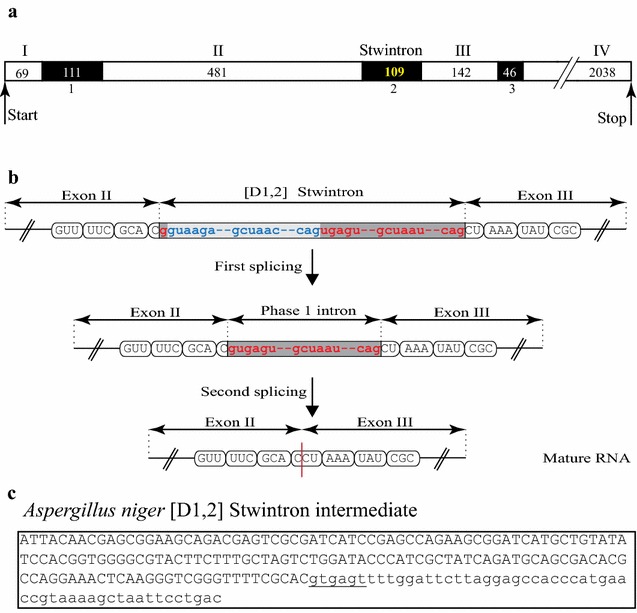
The [D1,2] stwintron of the putative lipase encoding gene of *A. niger* ATCC 1015. **a** Depicts the determined *A. niger lipS* intron–exon structure with the exact sizes of the exons and intervening sequences given: The stwintron is the second intervening sequence. **b** Schematically the structure of the stwintron that splits the CCU codon of Pro184, and the two consecutive splicing events necessary to remove it. Exonic sequences (in capitals) are grouped as the consecutive codons (Val181–Phe182–Ala183–Pro184–Lys185–Tyr186–Arg187). The internal intron is marked by the lighter grey bar; its 5′-donor-, lariat branch point domain- and 3′-acceptor sequences are printed in blue lettering. The external intron is marked by the darker grey bar; its 5′-donor-, lariat branch point domain- and 3′-acceptor sequences are printed in red lettering. **c** The existence of the splicing intermediate from which the internal intron has been removed by the first excision, has been confirmed experimentally (GenBank MF612153). The newly formed donor splice site sequence of the retained external intron, (5′-gugagu), is underlined

The four exon gene model is supported by Expressed Sequence Tag (EST) clone Asn_02874 from the related fungus *A. niger* (Accession DR703192), covering the stwintron position and the last intron position, 142 nt downstream. We independently verified the positions of the [D1,2] stwintron and the two other extant introns in the orthologue gene (miscalled locus ASPNIDRAFT_53020) by sequencing cDNA (GenBank MF612151) including the complete coding region encoding a peptide of 909 amino acids in *A. niger* ATCC 1015 (see Fig. 2a for the gene model). The 109-nt long [D1,2] stwintron splits the CCU codon for Pro184 in phase 1 (Fig. 2b). A 52-nt long internal intron (5′-GUAAGA—31-nt—GCUAAC—6-nt—CAG) would be nestled in the donor of a 57-nt long external intron between the first and second nt (5′-G|UGAGU—35-nt—GCUAAU—7-nt—CAG). We experimentally confirmed the expected splicing intermediate of the *A. niger* stwintron (GenBank MF612153) from which the predicted 52-nt internal intron was indeed removed (Fig. 2c).

### Identification of orthologues of the stwintron-containing gene

The peptide product of the stwintron-containing gene at locus AN7524 locally bears considerable similarity (46% identity, 56% similarity in a 182-residue subsequence) with a putative lipase/esterase (EC 3.1.1—carboxylic ester hydrolase) of 316 amino acids found in bacteria from the Burkholderiaceae family (RefSeq protein accession WP_054929686). This bacterial enzyme harbours a characteristic *alpha*/*beta* hydrolase fold 3 domain (Pfam07859) and is classified as belonging to the Hormone-sensitive_lipase_like_1 family of the *alpha*/*beta* hydrolase superfamily [14]. We therefore named the gene at locus AN7524, *lipS*, for putative lipase gene with stwintron. Note that the fungal protein is considerably larger than the bacterial *alpha*/*beta* hydrolases, with the Pfam07859 domain residing in the N-terminal half. Preliminary BLAST searches suggest the existence of a single, orthologue protein of substantial similarity beyond the *alpha*/*beta* hydrolase fold 3 domain in Pezizomycotina, Taphrinomycotina and all principal lineages of Basidiomycota albeit it is notably absent from Saccharomycotina, the third Ascomycota subphylum. In Ascomycota, there do not appear to be paralogues of high similarity like is the case for the family one Drug/H+ antiporter (DHA1), where we recently identified a genuine stwintron in some encoding genes in 73 species of Pezizomycotina from different classes [5].

46 amino acids from the central part of the sequences corresponding with the (bacterial) *alpha*/*beta* hydrolase fold 3 domain are encoded in the third exon between the [D1,2] stwintron and the downstream intron. The stwintron thus interrups the DNA encoding a structurally well-defined conserved domain, facilitating in depth analysis of its distribution. We collected hunderds of *lipS* orthologue genes in both phyla of Dikarya upon TBLASTN screening of the DNA databases with the *A. nidulans* protein as the query (data not shown). We noticed that in 52 Basidiomycota orthologue genes—a selection of genome-sequenced species representing all three subphyla—the stwintron position was not occupied despite the wealth of introns in certain taxa: *Basidioascus undulatus*, for instance, has 22 introns in its *lipS* orthologue gene and *Mrakia frigida*, 18. In the Taphrinomycotina subphylum (Ascomycota), the orthologue genes in *Taphrina* species have one intron at a unique position, 21 nt behind the start codon, while the gene is intronless in five other genome-sequenced species (including *Saitoella complicata*). On the other hand, both the stwintron position and the downstream third intron position in the DNA coding for the Pfam07859 domain were occupied in certain taxa of Pezizomycotina (as specified further below): the stwintron position thus appears to be specific to that subphylum. This was confirmed after manually verifying the intron–exon structure of some 150 *lipS* homologue genes in non-Dikarya species where some species harbour more than a dozen *lipS* paralogues (up to 27 paralogues in *Conidiobolus incongruus*).

**Fig. 3:**
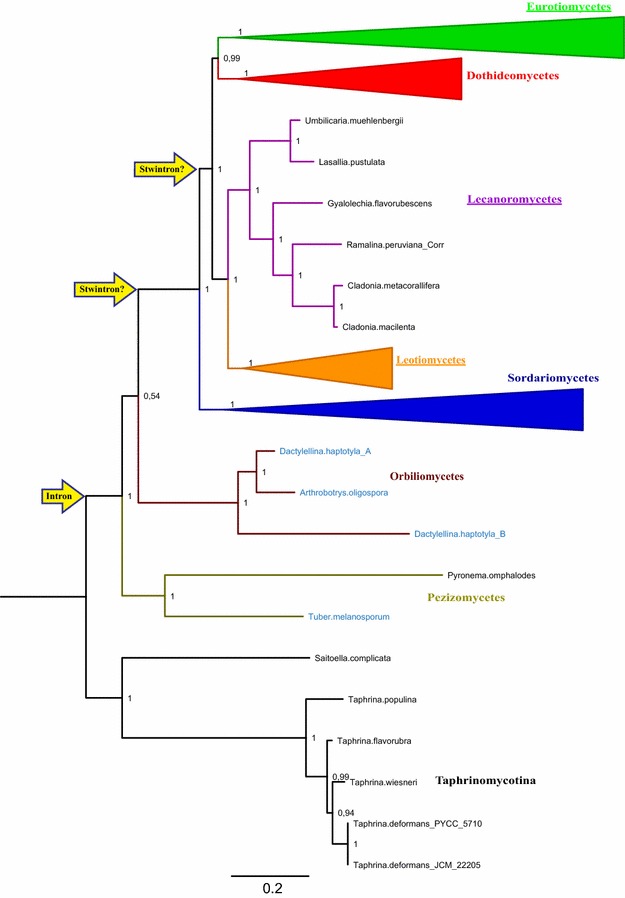
Maximum likelihood phylogeny of the LipS orthologue in the Ascomycota: Emergence of the [D1,2] stwintron. Taphrinomycotina proteins constitute the outgroup. Branch statistics are given as Approximate Likelihood Ratio Test values (0–1) at each node. The branches are color coded to distinguish the classes of Pezizomycotina; olive green: Pezizomycetes, auburn: Orbiliomycetes, blue: Sordariomycetes, green: Eurotiomycetes (NB. Including *A. nidulans*), red: Dothideomycetes, orange: Leotiomycetes, violet: Lecanoromycetes. The latter five classes belong to the Leotiomyceta super class. For the sake of simplicity, the clades for the four well-represented classes—Sordariomycetes, Eurotiomycetes, Dothideomycetes and Leotiomycetes—are collapsed. Classes in which the stwintron occurs are underlined. Species of Pezizomycetes and Orbiliomycetes, in which the stwintron position (second intron position in *Aspergillus*) is occupied by a standard intron in the orthologue gene, have their names printed in light blue. The two defining events in the formation of the stwintron are indicated by the yellow arrows at the left. The data do not allow to determine whether the stwintron emerged in Leotiomyceta before or after the divergence of the Sordariomycetes

Figure 3 shows a maximum likelihood tree of the putative lipase LipS protein in 292 species of Ascomycota with Taphrinomycotina proteins as the outgroup. The evolutionary relationships between the proteins in this analysis largely reflect fungal taxonomy. In species belonging to the early divergent classes of the Pezizomycetes and the Orbiliomycetes, the intron position where the [D1,2] stwintron resides in *Aspergillus*, is occupied by a standard U2 intron (the relevant species names are printed in light blue color in Fig. 3). In *Tuber melanosporum*, the U2 intron at the stwintron position is confirmed comparing the genomic sequence with that of EST clone SY0AAB55YD16 (Accession FP429675). Exceptionally, the *lipS* gene is duplicated in *Dactylellina haptotyla*; both genes have more than ten introns and include two very small exons of 3 nt. On the other hand, *Pyronema omphalodes* appears to have lost all but one intron, and lacks an intron at the stwintron position. Remarkably, the third intron position in *Aspergillus* (142 nt downstream of the stwintron position) is never occupied in species of the two early divergent classes of Pezizomycotina, suggesting it is exclusive to the Leotiomyceta superclass (see, e.g., [15], for a phylogeny-based classification of the Ascomycota). On the other hand, the first *Aspergillus* intron position (69 nt behind the ATG, splitting a His from a Tyr/Phe codon) is occupied in *T. melanosporum* and thus appears to be older.

The *lipS* [D1,2] stwintron is present across the classes of the Eurotiomycetes, Lecanoromycetes and Leotiomycetes (underlined in Fig. 3) and would thus be older than most stwintrons we have uncovered to date. In these Leotiomyceta, all three intron positions that occur in the *A. nidulans* gene (see above) are conserved although certain lineages appear to have lost intronic sequences (as specified further below). On the contrary, in the classes of the Sordariomycetes and the Dothideomycetes, the positions of stwintron and the third *Aspergillus* intron are not occupied. Sordariomycetes and some taxa of Dothideomycetes (Botryosphaeriales and Dothideales, e.g.) still harbor an intron at the first *Aspergillus* intron position but in Pleosporales, the *lipS* intron–exon structure has been reset completely with three introns appearing at new positions. In most Capnodiales, the *lipS* gene is intronless. Importantly, we could not find Leotiomyceta species in which the *lipS* stwintron position is occupied by a standard canonical intron, hence, this latter situation is unique to Pezizomycetes and Orbiliomycetes species. The LipS phylogeny thus strongly suggests that the [D1,2] stwintron has emerged at the position of a pre-extant canonical “host” intron, currently serving as the stwintron’s external intron.

**Fig. 4:**
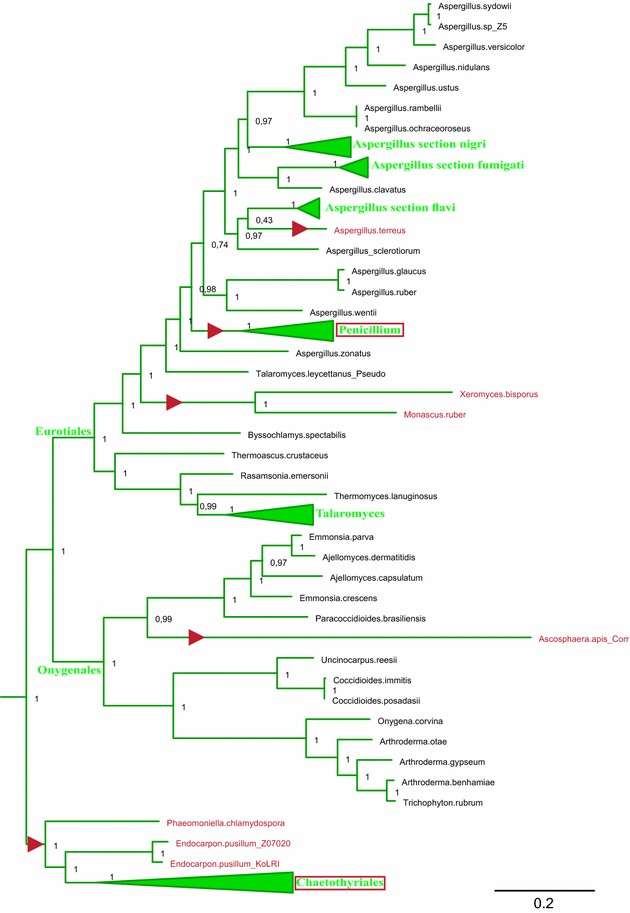
Maximum likelihood phylogeny of LipS: Instances of stwintron loss in Eurotiomycetes. A subtree of the phylogenetic analysis depicted in Fig. 3, is shown in detail to highlight the loss of the stwintron from taxa of Eurotiomycetes. Class-specific color coding is the same as in Fig. 3. Species that have lost the stwintron from their *lipS* gene have their name printed in red lettering. For the sake of simplicity, we have collapsed groups of related fungi that behave identically with respect to stwintron presence/absence. All the species in the collapsed clades for the Chaetothyriales order and the *Penicillium* genus have no intronic sequences at the stwintron position (second intron position in *Aspergillus*) and the taxon names are therefore marked with red boxes to highlight stwintron loss. Independent events of stwintron loss are also indicated by red triangles on the directly preceding branches (NB. The position of the triangles does not correspond with the exact time point at which stwintron loss has taken place)

**Fig. 5:**
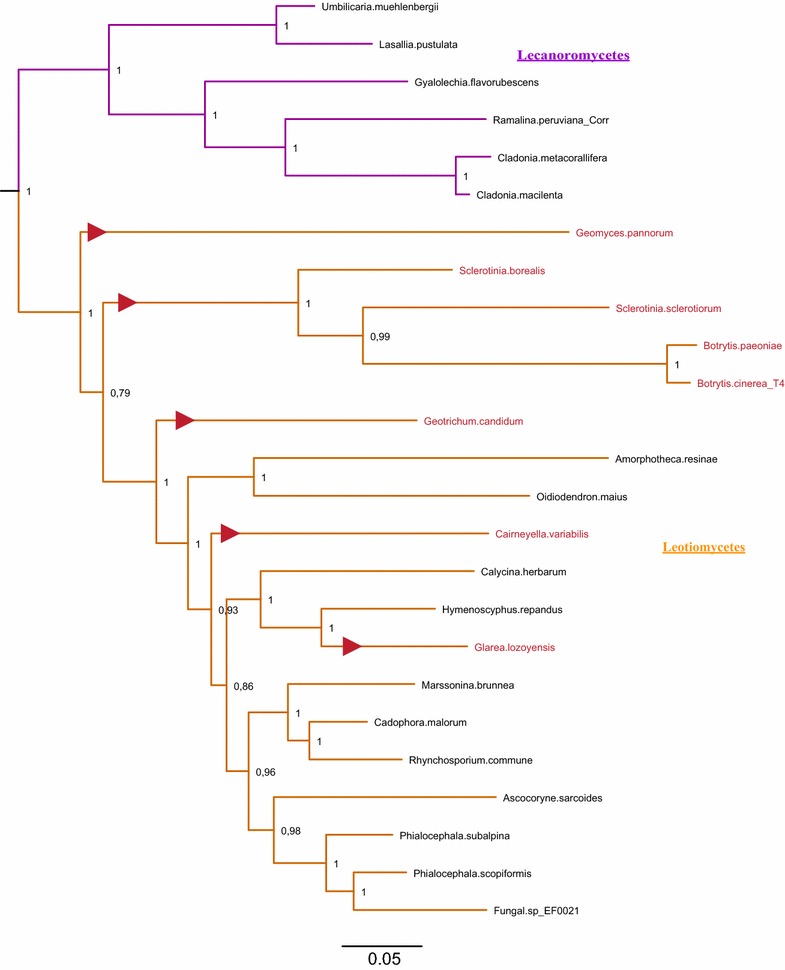
Maximum likelihood phylogeny of LipS: Instances of stwintron loss in Leotiomycetes. A subtree of the phylogenetic analysis depicted in Fig. 3, is shown in detail to highlight the loss of the stwintron from taxa of Leotiomycetes. Class-specific color coding is the same as in Fig. 3. Species that have lost the stwintron from their *lipS* gene have their name printed in red lettering. Independent events of stwintron loss are also indicated by red triangles on the directly preceding branches (NB. The position of the triangles does not correspond with the exact time point at which stwintron loss has taken place)

Figures 4 and 5 show the clades of species that include the stwintron in more detail, to highlight instances of stwintron loss in Eurotiomycetes and Leotiomycetes, respectively (fungi that lost the *lipS* stwintron are marked in red lettering in both figures). In the Eurotiomycetes (Fig. 4), the stwintron is completely absent from genome-sequenced species in the orders of the Chaetothyriales, Verrucariales and Phaeomoniellales. In the Eurotiales order, the stwintron is completely absent from the genus of *Penicillium*, the sequenced *Monascus* and *Xeromyces* species as well as from *Aspergillus terreus* while in the Onygenales, *Ascosphaera apis* lacks it. Thus the stwintron has been lost with the bordering exons fused at at least five independent occasions in the Eurotiomycetes. Meanwhile, the stwintron is absent in eight species of Leotiomycetes (Fig. 5). The latter include all species from the family of the Sclerotiniaceae in our analysis, along with *Pseudogymnoascus pannorum*, *Glarea lozoyensis*, *Cairneyella variabilis* and “*Geotrichum candidum* 3C” (NB. The *G. candidum* 3C genome sequences (WGS Master Accession JMRO01000000) strongly suggest that they are from a species of Leotiomycetes rather than from a species of Saccharomycetales: All Saccharomycetales lack a *lipS* orthologue, see above). It is remarkable that in the majority of the above cases of stwintron loss, the intron at the third *Aspergillus* position has also disappeared, suggesting simultaneous loss. Only in *A. terreus*, *A. apis* and “*G. candidum* 3C”, the downstream intron is retained, while we found the reverse situation (i.e., only the intron at the third *Aspergillus* position lost) in *Marssonina brunnea* and *Calycina herbarum*.

### Reappraisal of the *bioDA* stwintron

In a previous stwintron study, we had identified another potentially ancient stwintron in *bioDA*, a structural gene of D-biotin biosynthesis encoding a bifunctional protein exhibiting dethiobiotin synthetase (EC 6.3.3.3—BioD) and *S*-adenosyl-l-methionine:8-amino-7-oxononanoate aminotransferase (EC 2.6.1.62—BioA) activities (cf. [16]). We showed that a [D2,3] stwintron occurs at the position of the first (standard) intron in *A. nidulans bioDA* in most (31) species of the Sordariomycetes class and the two species of the Botryosphaeriales order of the Dothideomycetes class for which genome sequences were available at the time. In a phylogenetic analysis ([3]; see Supplementary Fig. S4 thereof), the two Botryosphaeriales proteins clustered with those from the Sordariomycetes and not with those from the other Dothideomycetes (24 species). Remarkably, in *Nectria haemotococca* (formerly known as *Fusarium solani f. sp. pisi*) a standard intron occupies the position of the stwintron in the other Sordariomycetes, including in other *Fusarium* species. Since the spring of 2013, hundreds of new Pezizomycotina genome sequences have been added to publicly accessible databases. We took the opportunity to update our analysis of the *bioDA* stwintron, to compare defining aspects of its evolution with those of the (new) stwintron in the putative lipase *lipS* gene (see above).

We screened the databases with TBLASTN using the *A. nidulans* protein (protein accession number ACR44943) as the query and collected hundreds of fungal *bioDA* genes for which we manually deduced the intron–exon structure and the cognate protein (results not shown). From the updated collection from non-Pezizomycotina taxa, we can now confirm that the intron position at which the stwintron occurs (in Sordariomycetes and Botryosphaeriales, see below) is deeply rooted in fungal evolution (see, e.g., [17], for the taxonomy of the fungal kingdom). This intron position is occupied by a standard U2 intron in species of Mucoromycotina, Mortierellomycotina, Entomophthoromycotina and Chytridiomycota albeit absent from *Rhizophagus irregularis* (Glomeromycota) and Blastocladiomycota (results not shown). Remarkably, in both sequenced strains of *Batrachochytrium dendrobatidis*, the donor of this intron is a non-canonical 5′-GAAAGA. (NB. *bioDA* is absent from Microsporidia, Cryptomycota, Neocallimastigomycota and Kickxellomycotina). Within Dikarya, this intron position is conserved across the three subphyla of the Basidiomycota. We also found the stwintron position occupied by a standard intron in three species of Saccharomycetales (*Saprochaete clavata*, *Lipomyces starkeyi* and *Sugiyamaella xylanicola*) amongst those that (still) have the *bioDA* gene. On the other hand, there is no intron present at that position in the seven genome-sequenced species of Taphrinomycotina that have the *bioDA* gene (results not shown).

**Fig. 6:**
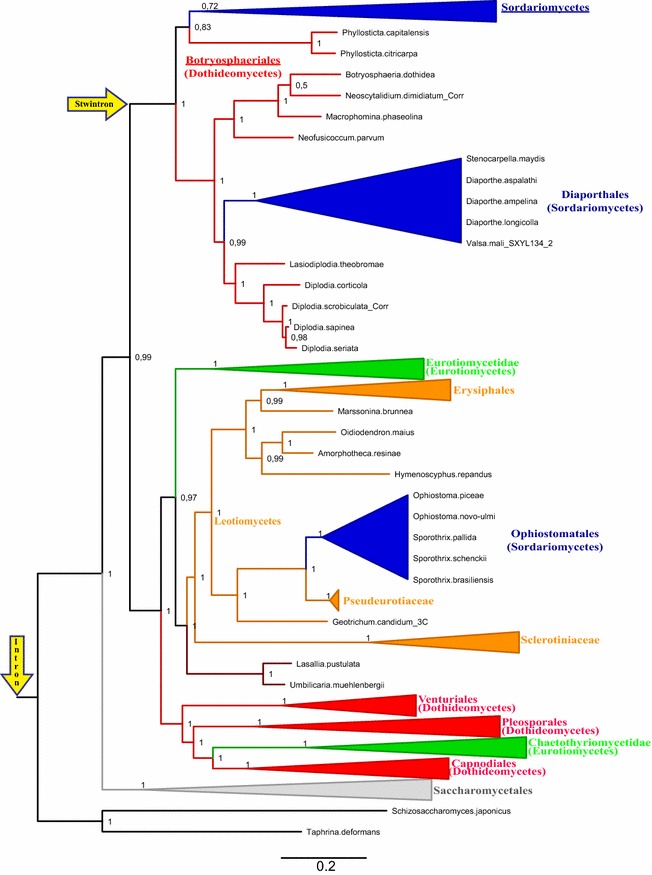
Maximum likelihood phylogeny of the BioDA protein in the Ascomycota: Emergence of the [D2,3] stwintron. Taphrinomycotina proteins constitute the outgroup. Branch statistics are given as Approximate Likelihood Ratio Test values (0–1) at each node. The branches are color coded to distinguish classes of Pezizomycotina, as described in the legend to Fig. 3. Some fungal taxa are collapsed, to the level of whole classes for most of the Pezizomycotina. Taxa in which the stwintron occurs—Sordariomycetes and Botryosphaeriales—are underlined. The two groups of Sordariomycetes that stand out for the absence of intronic sequences at the *bioDA* stwintron position—members of two families of Diaporthales and five species of the order of the Ophiostomatales, respectively—are cartooned (blue) rather than collapsed. The branch where the stwintron has emerged at the position of an ancient intron is indicated by the upper yellow arrow. The latter “host” intron occurs in many species of non-Dikarya (see “Results” section): To indicate its kingdom-wide existence, the lower yellow arrow points at the origin of the tree. Note that a Maximum Likelihood phylogeny based on an alignment calculated using more stringent parameters (similarity matrix BLOSUM62) suggested that the Botryosphaeriales clade is paraphyletic to the (main) Sordariomycetes clade

We carried out a phylogenetic analysis of 298 Ascomycete BioDA proteins which we had deduced manually from our *bioDA* gene collection. Taphrinomycotina serve as the outgroup and the Saccharomycotina proteins constitute a sister clade to the Pezizomycotina (Fig. 6). Unlike for the putative lipase LipS, the evolutionary relations between Pezizomycotina BioDA proteins do not conform the standard fungal taxonomy as, for instance, the classes of the Sordariomycetes (cf. [18]), Dothideomycetes (cf. [19]) and Eurotiomycetes (cf. [20]) do not behave as monophyletic.

However, the fate of the relevant intron position in Pezizomycotina *bioDA* appeared more conventional. The [D2,3] stwintron occurs in all orders and in most species of Sordariomycetes and in Botryosphaeriales (Dothideomycetes), which cluster together in one clade (Fig. 6) albeit not all species in this clade have the *bioDA* stwintron. In the other Dothideomycetes (including, the Pleosporales and Capnodiales orders) as well as in the Eurotiomycetes class, a standard U2 intron is present at the *bioDA* stwintron position. On the other hand, in the Leotiomycetes class and in the Umbilicariomycetidae family (NB. *Lasallia pustulata* and *Umbilicaria muehlenbergii*; the other genome-sequenced Lecanoromycetes do not have *bioDA*) that ancient intron position is lost.

The *bioDA* genes from some of the species of the Ophiostomatales order of the Sordariomycetes appear to originate from a Leotiomycetes ancestor, and consequently do not harbour an intron at the stwintron position. This is probably the strongest indication to lateral transmission of *bioDA* between not directly related taxons of Pezizomycotina. This particular *bioDA* gene transfer is likely to have occurred recently as Ophiostomatales species *Rafaelea quercivora* and *Leptographium procerum* do harbour the *bioDA* stwintron and their BioDA proteins cluster together with those of all other Sordariomycetes orders. Such an event is not exceptional for structural genes of biotin biosynthesis: The acquisition of *bioA* and *bioD* genes from an unspecified bacterial source is well documented for *Saccharomyces cerevisiae* [21].

**Fig. 7:**
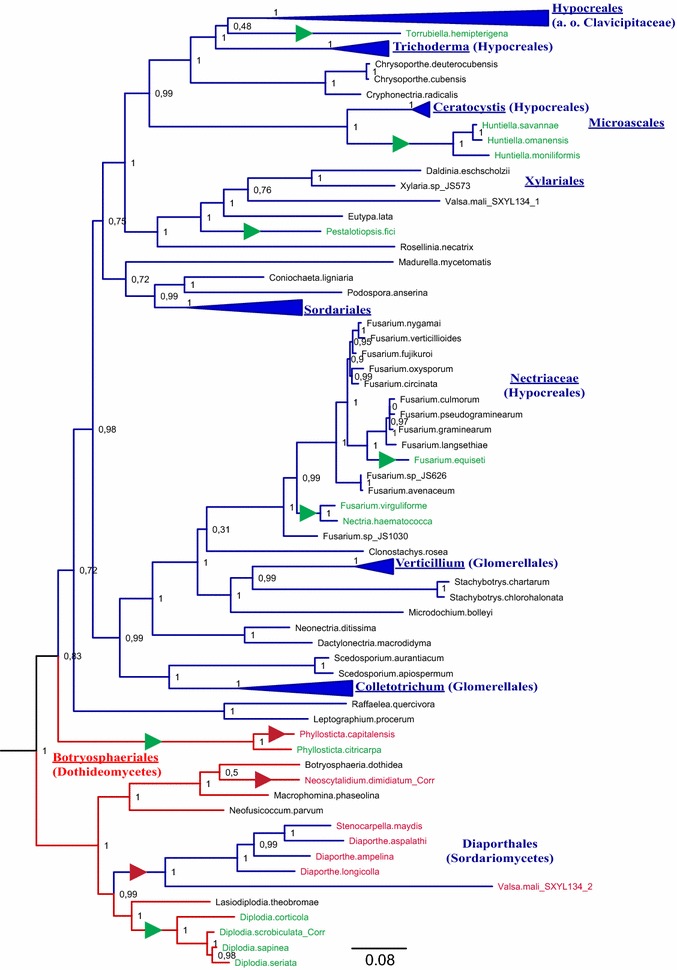
Maximum likelihood phylogeny of the BioDA protein: Instances of stwintron loss. A subtree of the phylogenetic analysis depicted in Fig. 6, is shown in detail to highlight the loss of the stwintron from taxa of Sordariomycetes and Botryosphaeriales. Class-specific color coding is the same as in Fig. 3. Species that have lost the internal intron from the stwintron in their *bioDA* gene (retaining a standard intron at the stwintron position) have their name printed in green lettering. For the sake of simplicity, we have collapsed groups of related fungi that behave the same with respect to the stwintron. Independent events of internal intron loss are also indicated by the green triangles on the directly preceding branches. (NB. The position of the triangles does not correspond with the exact time point at which intron loss has taken place). The complete absence of intronic sequences at the stwintron position from the genes encoding the BioDA proteins in the separate Diaporthales clade and in two of the Botryosphaeriales (7 proteins in red lettering) may have occurred either in one or in two consecutive events of intron loss. Complete stwintron loss was indicated by a red triangle (as in the legend to Fig. 4). We annotated the tree for the two-step process in the case of *P. capitalensis*, first losing the internal intron in an ancestor shared with *P.citricarpa* (green triangle). The subsequent loss of the standard intron that remained after the first event (red triangle) resulted in the complete absence of intronic sequences from *P. capitalensis bioDA*

Figure 7 shows the clade of species that harbour the *bioDA* stwintron in more detail. Unlike the situation with the putative lipase stwintron, most instances of stwintron loss from the *bioDA* gene result in the presence of a standard intron at the same position (relevant species names in green in Fig. 7). Loss of the internal intron has happened at at least five independent instances in the clade that constitute the large majority of the Sordariomycetes species, in three different orders—Hypocreales, Xylariales and Microascales—including the instance previously observed in *N. haematococca bioDA*. In Botryosphaeriales, we observe the loss of the internal intron in five of the eleven species investigated, corresponding to two independent events. Furthermore, in two Botryosphaeriales species, *Phyllosticta capitalensis* and *Neoscytalidium dimidiatum*, there are no intronic sequences left at the stwintron position (species names in red in Fig. 7). The current phylogenetic analysis suggests that the loss of the complete stwintron in *N. dimidiatum* happened in one event, like in all cases of stwintron loss from the *lipS* gene (see above; Figs. 4, 5). On the contrary, the stwintron sequences may have been lost from *P. capitalensis bioDA* in two consecutive intron loss events, since its close relative *P. citricarpa* (same genus) has retained a standard intron at the stwintron position.

A number of species from the Diaporthales order—more specifically, the members of the Diaporthaceae and Valsaceae families—also have a *bioDA* gene from which the stwintron is completely absent (relevant species names in red in Fig. 7). The genes encoding the five clustered Diaporthales proteins appear to derive from a Botryosphaeriales taxon diverged from the main Sordariomycetes branch, the latter including species of another Diaporthales family, the Cryphonectriaceae (*Chrysoporthe cubensis*, *Chrysoporthe deuterocubensis* and *Cryphonectria radicalis*). Interestingly, *Valsa mali* (WGS Master accessions JUIY00000000 and JUIZ00000000) has two *bioDA* genes: The gene that encodes the protein in the separate Diaporthales clade has lost all stwintron sequences while the second gene, which carries a complete stwintron, is more related with Xylariales genes. Our current phylogenetic analysis suggests that the absence of all *bioDA* stwintron sequences from the five species of Diaporthales is due to their loss in one event (like in *N. dimidiatum*, see above) since one of the species in the divergent branch of Botryosphaeriales, *Lasiodiplodia theobromae*, (still) harbours a stwintron.

## Discussion

In this paper, we evidence a new [D1,2] stwintron in a putative lipase gene in *A. nidulans* and *A. niger*. It resides within DNA that encodes a well conserved *alpha*/*beta* hydrolase fold domain allowing deep phylogenetic analysis to trace the emergence and evolution of this stwintron. In parallel, we monitored the fate of the ancient stwintron in the bifunctional biotin biosynthesis gene *bioDA*, previously characterised in *Trichoderma reesei* (cf. [3]).

The fungal BioDA phylogeny does not conform to the accepted taxonomy, however, the groups of fungi wherein the [D2,3] stwintron occurs cluster together to form a clearly defined clade (Fig. 6). The [D2,3] stwintron in most Sordariomycetes and in various Botryosphaeriales occupies the same position as a standard U2 intron in other Dothideomycetes orders and in the Eurotiomycetes. This is a very ancient intron position which appears to predate the emergence of Dikarya. Therefore, our results leave little doubt that the [D2,3] stwintron emerged by the appearance or insertion of an “internal” intron between the second and third nt of the donor sequence of a pre-extant “host” intron in the *bioDA* gene. It is possible that the internal intron has “matured” within the host intron, a process we have previously termed “stwintronisation” [3] as it resembles the “intronisation” of exonic sequences [22]. Alternatively, intron insertion may have taken place abruptly in one event, during the repair of double strand DNA breaks by the non-homologous end joining machinery [23]). These two modes of stwintron formation by the appearance of a new intron in one of the terminal splice site sequences of a pre-extant intron, are consistent with two proposed mechanisms of intron gain from an endogenous origin (reviewed by [24]).

Phylogenetics of the putative lipase LipS (Fig. 3) suggests that the [D1,2] stwintron present in certain taxa of the Leotiomyceta superclass was formed in essentially the same way, at the position of an older, canonical U2 intron. This latter has survived as a standard intron in the early divergent classes of Pezizomycetes and Orbiliomycetes while the external intron of the stwintron in Leotiomyceta derives from it. From our data collection, it is not clear whether the *lipS* stwintron appeared before or after the divergence of the Sordariomycetes from the other Leotiomyceta lineages. Sordariomycetes lack intervening sequences at the *lipS* stwintron position. This situation could result from either the loss of the predecessor standard intron before the first appearance of the stwintron, or the class-specific loss of the complete stwintron after its formation in a common ancestor of all Leotiomyceta.

Such a “stwintron loss” event appears to have occurred at several occasions in the evolution of the *lipS* orthologous gene in the Leotiomyceta superclass, including other events that have led to the absence of the stwintron from all species from the class of the Dothideomycetes, all species from the superorder of the Chaetothyriomycetidae (Eurotiomycetes class) and all species from the *Penicillium* genus (Eurotiales order of the Eurotiomycetes class). Each of these instances of stwintron loss appears to coincide with the absence or the loss of the intron at the third position in the *Aspergillus lipS* gene, 142 bp downstream of the stwintron. Indeed, the simultaneous loss of separate intervening sequences may have occurred in all but four instances (all four apparently involving just one species—*A. terreus*, *A. apis*, *C. variabilis* and “*G. candidum* 3C”) (Figs. 4, 5). On the other hand, in Sordariomycetes, the loss of the stwintron from the *bioDA* gene has resulted in the retention of a standard U2 intron in all but one instance (i.e., that involving the separate clade of five Diaportales, see above) (Fig. 7). It would thus appear that in most Sordariomycetes *bioDA*, the event(s) leading to the emergence of an internal intron was/were reversed to return to the primordial situation.

Despite this rather striking difference between the two ancient stwintrons, all observed instances of accurate intron loss are compatible with one single mechanism, involving reverse transcription of spliced transcripts and homologous recombination of cDNA at the genome locus (e.g., [25]; reviewed by [26]). This mechanism allows the simultaneous disappearance of two introns (bordering the same exon) but also the removal of the internal intron from a stwintron, if the stwintron splicing intermediate rather than the mature messenger served as reverse transcriptase template. It is tempting to speculate that the longevity of the stwintron splicing intermediate in the nucleus—dependent of the relative excision rates of the internal- and the external introns and the export of the messenger from the nucleus—is an important factor in the reversal of a stwintron into a standard intron at the same position. We have proven recently that alternative removal of terminally overlapping internal introns from an alternatively spliced [D1,2]/[A2,3] stwintron leads to discordant introns in orthologue genes [5], suggesting that the events we observed in the current study of the *bioDA* stwintron are not exceptional.

## Conclusions

Molecular phylogeny of the peptide product was used to monitor the emergence and disappearance of an ancient spliceosomal twin intron in the transcripts of two neatly defined fungal genes, encoding well conserved proteins. These stwintrons occur across complete fungal orders and classes and both defining events can be explained with extant models for intron insertion and -loss. We thus demonstrated that stwintrons can serve as model systems to study spliceosomal intron evolution.

## Methods

### Nucleic acid isolation

*Aspergillus nidulans* ATCC 48756 (R21) and *A. niger* ATCC 1015 were used to confirm the existence of a stwintron in the transcript of their orthologue genes for a putative lipase characterised by the well-conserved *alpha*/*beta* hydrolase fold 3 domain (Pfam07859). Standardised medium compositions are described elsewhere [10]. Fungal biomass was generated in 500-mL Erlenmeyer flasks with 100 mL of complete medium seeded with vegetative spore inoculum, in a rotary shaker (Infors HT Multitron) at 200 rotations per min. 16 h after inoculation, mycelia were harvested by filtration, thoroughly washed with distilled water, and subsequently frozen and ground to powder under liquid nitrogen. For the extraction of genomic DNA and total RNA from the mycelial powder, Macherey–Nagel NucleoSpin kits (NucleoSpin Plant II and NucleoSpin RNA Plant) were used.

### Reverse transcription PCR (RT-PCR)

Reverse transcription was performed with 1 µg of total RNA as the template and Oligo(dT) as a primer in a 20 µL reaction volume using the RevertAid First Strand cDNA Synthesis Kit (Thermo Scientific). Subsequent PCR reactions were done in a 25 µL volume containing 4 µL of the single strand cDNA, using gene-specific oligonucleotides (Additional file 1: Table S1) as primers and DreamTaq DNA Polymerase (Thermo Scientific). Cycling conditions after initial denaturation at 95 °C for 2 min were: 35 cycles of 95 °C for 30 s, 60 °C for 1 min, and 72 °C for 1 min, followed by one post-cyclic elongation at 72 °C for 5 min. Amplified fragments were resolved in native agarose gels.

To confirm the existence of the predicted stwintron splicing intermediates, we used the same approach as previously with PCR primer pairs that do not amplify off cDNA template from fully spliced mRNA. This strategy usually yields two fragments of defined sizes of which the smaller one corresponds to the splicing intermediate and the bigger one, to primary transcript. All experiments were done in duplicate, starting with biomass from two independent liquid cultures.

### cDNA sequencing

Double strand cDNA was gel-purified (NucleoSpin Gel and PCR Clean-up, Macherey–Nagel) and cloned (pGEM-T Easy Vector System I, Promega). Plasmid DNA was isolated using the NucleoSpin Plasmid EasyPure kit (Macherey–Nagel). Three independent clones were sequenced over both strands using universal primers hybridising to the vector (MWG-Biotech AG, Ebersberg, Germany) or gene-specific oligonucleotide primers where appropriate. cDNA sequences were deposited at GenBank under accession numbers MF612150–MF612153.

### Phylogenetic analyses

For the analysis of the putative lipase orthologue, 292 proteins were aligned with Multiple Alignment using Fast Fourier Transform (MAFFT; version 7) [27], applying the E-INS-i algorithm and a BLOSUM 62 similarity matrix. The MAFFT alignment was trimmed with Block Mapping and Gathering with Entropy software (BMGE version 1.12) [28] using BLOSUM 30 and a block size of 5. A Maximum Likelihood tree was then calculated on-line from the curated alignment by PhyML 3.0 [29] using the WAG substitution model.

For the study of the bifunctional BioDA protein, 298 proteins were aligned using a BLOSUM 45 similarity matrix in the MAFFT E-INS-i module. The alignment was BMGE trimmed using BLOSUM 55 and a block size of 5, before a Maximum Likelihood phylogeny was inferred using the WAG substitution model.

For each tree, approximate Likelihood Ratio Tests provide statistical branch support [30]. The trees were drawn from the Newick output of PhyML with the FigTree program (http://tree.bio.ed.ac.uk/software/figtree) and further annotated with Adobe Illustrator.

## Additional files

**Additional file 1: Table S1.** Oligonucleotide primers used in this study.

**Additional file 2: Table S2.** Sequences used for the lipase phylogeny (Fig. 3).

**Additional file 3: Table S3.** Sequences used for the BioDA phylogeny (Fig. 6).

## Abbreviation

nt: nucleotides
