# Supplementary material for: Emergence and loss of spliceosomal twin introns
Source: Fungal Biol Biotechnol. 2017 Oct 6;4:7. doi: 10.1186/s40694-017-0037-y (PMC5639578; doi:10.1186/s40694-017-0037-y)
Supplement: Supplementary file 1 — Additional file 1: Table S1. Oligonucleotide primers used in this study. [file 40694_2017_37_MOESM1_ESM.doc]

**Additional file 1**

**Table S1: Oligonucleotide primers used in this study**

Oligonucleotide primers for RT-PCR verification of [D1,2] stwintron splicing intermediates

|  | **Locus** | **Primer** | **Sequence (5’-)** |
| --- | --- | --- | --- |
| *Aspergillus nidulans* | AN7524 | P1_F | ATTGCGAGGCGAGTGGATTG |
| *Aspergillus nidulans* | AN7524 | P2_R | GCGGTAGTCGGCTTCTATTC |
| *Aspergillus nidulans* | AN7524 | P3_R | TAGCGGCAGTCCTTGGTCAC |
| *Aspergillus niger* | ASPNIDRAFT_53020 | P1_F | GTGGATTGAGATGAAGGATG |
| *Aspergillus niger* | ASPNIDRAFT_53020 | P2_R | GCGCCAGGCGATATTTAGCTG |
| *Aspergillus niger* | ASPNIDRAFT_53020 | P3_R | AGGGAGAAATTAAAATAGCAC |

**Oligonucleotides for cDNA cloning and** sequencing

|  | **Locus** | **Primer** | **Sequence (5’-)** |
| --- | --- | --- | --- |
| *Aspergillus nidulans* | AN7524 | AN7524_F1 | GCAATGAGCATCCTTGACG |
| *Aspergillus nidulans* | AN7524 | P3_R | TAGCGGCAGTCCTTGGTCAC |
| *Aspergillus nidulans* | AN7524 | AN7524_F2 | GAATAGAAGCCGACTACCGC |
| *Aspergillus nidulans* | AN7524 | AN7524_R | AGGTATCAATCTAGCTCCAA |
| *Aspergillus niger* | ASPNIDRAFT_53020 | 53020_ F1 | GGTCATCTTTACCTCCTCTAG |
| *Aspergillus niger* | ASPNIDRAFT_53020 | P3_R | AGGGAGAAATTAAAATAGCAC |
| *Aspergillus niger* | ASPNIDRAFT_53020 | 53020_ F2 | CAGCTAAATATCGCCTGGCGC |
| *Aspergillus niger* | ASPNIDRAFT_53020 | 53020_ R | CGACAACCACTGACTGCCAAC |
